# Model Test on Acoustic Emission Monitoring of Loess Slope Failure

**DOI:** 10.3390/s24216851

**Published:** 2024-10-25

**Authors:** Xiaoyu Yang, Xiaohui Sun, Shengdi He, Yanrong Li

**Affiliations:** Department of Earth Sciences and Engineering, Taiyuan University of Technology, Taiyuan 030024, China; yangxy112024@163.com (X.Y.); sunxh17@126.com (X.S.); heshengdi@tuyt.edu.cn (S.H.)

**Keywords:** loess collapse, waveguide system, model experiment, AE

## Abstract

The three stages of loess collapse are characterized by notable concealment and sudden onset due to the sudden nature of loess collapse and the prolonged duration of the peristaltic deformation stage. Traditional displacement monitoring methods struggle to detect early signals of instability and failure, leading to poor timeliness in disaster warnings. This project begins by examining non-force field information related to the loess collapse process. It focuses on acoustic emission monitoring and employs model tests to identify effective waveguide rods for monitoring loess collapse. Additionally, the project investigates the evolution anomalies of acoustic emission parameters before and after loess collapse failure, aiming to establish early warning criteria for loess collapse based on acoustic emission. This work provides a theoretical basis for monitoring and early warning of loess collapses. This study evaluates five parameters of the active waveguide system: sensor installation method, filling material, waveguide rod wall thickness, outer wrapping material, and outer wrapping wall thickness. The densities of the filler materials were tested using the optimal parameters derived from the tests to identify the best configurations for active acoustic emission (AE) waveguide systems suitable for monitoring loess collapse. Subsequently, a one-sided connected loess collapse model was employed for indoor tests, integrating real-time AE monitoring with the active waveguide method. This model facilitates the exploration of AE response characteristics during loess collapse and the analysis of destructive forms of loess collapse and time-sequence evolution of AE ringing counts throughout the deformation and destruction process. Results indicate that using filler materials with high elasticity modulus, high compactness, and low Poisson’s ratio, along with thin outer wrapping and waveguide rod walls, leads to strong AE signals. As deformation damage of loess collapse intensifies, the number of AE ringing counts notably increases. A rapid rise in cumulative ringing counts can indicate a “sudden increase”, or the b-value may stabilize, providing precursor information for loess collapse.

## 1. Introduction

Loess collapse is a common geohazard that causes considerable loss of life and property annually, particularly in the Loess Plateau region of China [1,2,3]. Slope monitoring and early warning are crucial methods for disaster prevention and mitigation. Currently, geological hazard monitoring methods mainly include four categories: environmental factor monitoring, surface deformation monitoring, underground deformation monitoring, and physical and chemical monitoring [4,5]. The deformation characteristics and precursors of geological disasters can be indirectly obtained by analyzing the relationship between rainfall and geological disasters [6]. Surface and underground deformation monitoring gathers data by measuring relative displacement changes at the surface and underground, enabling real-time monitoring of the deformation characteristics of geological hazards. Physical and chemical monitoring can provide information such as infrasound and stress changes associated with the deformation process of geological disasters. At present, geological disaster monitoring equipment exhibits diverse characteristics, and recent advancements in equipment performance and monitoring accuracy have notably improved. In particular, the emergence of “space–space–ground” integrated geological disaster monitoring technology has notably promoted the capabilities for early identification, monitoring, and warning technology of geological disasters.

Loess collapse is characterized by its high suddenness, with no evident displacement or deformation occurring before instability, resulting in a relatively short disaster response time. Traditional geological disaster monitoring mainly monitors the stress and displacement of loess slopes, providing effective feedback for slope geological disasters that exhibit clear creep deformation. However, for sudden loess collapses, immediately capturing displacement precursor information and providing targeted early warnings is challenging. Previous studies have largely concentrated on pre-damage early warnings through slope deformation analysis [7,8,9]. However, pre-displacement signals indicating slope failure may not be detectable using these traditional methods. While loess collapse does not exhibit evident deformation before failure, microfractures begin to form during the early stages of slope deterioration. This condition leads to the release of energy in the form of elastic waves, known as acoustic emission (AE), due to the movement or fracturing of soil particles. Therefore, AE monitoring can be employed to capture early signals of loess collapse. AE signals can be used to analyze the time–frequency domain patterns of primary and secondary fissure emergence, extension, and penetration within a geotechnical body. This analysis provides insights into the characteristics associated with the different states of structural stability, the onset of damage, and the progression of failure in a geotechnical body [10,11,12]. The application of AE technology to the field of geotechnical engineering began in the 1960s. Numerous scholars conducted extensive AE tests to investigate the rock mass fracture process, exploring the relationships among key AE parameters, fractal characteristics, B-values, and the rock fracture process [13,14]. For example, Zhang [15] et al. established a statistical relationship between the energy ratio of low and high main frequency AE waveforms and the peak strength of rock samples through rock tensile tests. Shiotani et al. [16] installed multiple AE sensors in waveguide rods at specific distances and used cement mortar embedded in the rock slope to monitor slope stability. However, research on the AE response characteristics of soil remains in its early stages due to the relatively small energy of AE signals during soil deformation and failure, substantial energy loss of acoustic signal during signal propagation, and considerable background noise interference. Koerner et al. [17], were among the first to apply AE technology to monitor soil deformation and failure processes, examining the variation characteristics of AE signals in noncohesive and cohesive soils under different confining pressures and moisture contents. Tanimoto et al. [18] further investigated the AE characteristics of sand subjected to triaxial compression.

Two types of AE waveguide systems are currently available for slope warning: active and passive waveguides. Passive waveguides are commonly utilized for AE monitoring of rocky slopes. In a study conducted by an Italian scholar [19], waveguide rod grout was inserted into rocky slopes to monitor their stability. The study demonstrated that AE monitoring can effectively detect deformation in rocky slopes and that the AE data are sensitive to even minor changes in stress. However, when monitoring soils with AE, active waveguide systems are typically preferred due to the low level of AE signals and considerable attenuation during propagation [18]. The signals primarily arise from the mutual friction between particles of the fill material [20,21]. Smith (2017) [21] conducted indoor model tests in which an active waveguide was inserted into a large soil sample during shearing, demonstrating that the system could relate measured AE parameters with slope movement velocity, providing early warning information before destabilization. Similarly, Berg (2018) [22] used an active waveguide system to monitor slopes, establishing a quantitative relationship between AE parameters and slope movement velocity. This system can be used for early warning. However, collecting sufficient deformation and displacement data to establish the relationship between the AE signal and the moving speed is challenging for loess collapse geohazards. This difficulty is attributed to the weak deformation before damage and its transient nature. Therefore, the active waveguide system suitable for loess collapse must be explored, and the co-evolution relationship between loess collapse development and AE processes must be established.

Among existing active waveguide models, some scholars have explored the properties of filling materials, waveguide rod materials, and wrapping materials. According to Spriggs (2004) [23], gravel produces a higher number and amplitude of AE events compared to sand as fill materials. Steel waveguide rods are frequently used in soil monitoring because they exhibit lower attenuation of high-frequency wave propagation along steel tubes than fine-grained soil media [24]. In indoor tests, peripheral wrapping was performed using 2 mm thick HDPE geomembranes, 2.5 mm thick rubber tubing, or no wrapping to fill directly into the soil [25,26,27]. However, current research on waveguide modeling lacks a comprehensive system to determine whether effective results can be achieved in AE monitoring of Maran loess.

This study has three objectives: (1) to assess the feasibility of waveguide systems in applications related to loess collapse; (2) to analyze the factors affecting the strength of AE signals from active waveguide systems; and (3) to evaluate the effectiveness of AE signature parameters as warnings for loess collapse monitoring. Five orthogonal tests were conducted using the three-point bending test method to measure AE signal strength across various sensor installation methods, filler materials, waveguide rod wall thicknesses, wrapping materials, and outer wrapping wall thicknesses. An indoor collapse test system was subsequently designed to verify the reliability of the active waveguide system in monitoring loess collapse, simulating the entire process from stress concentration and local damage to final damage. An AE monitoring system, along with other measuring equipment, was used to investigate the AE parameters generated during the loess collapse, providing strong support for AE monitoring and early warning of such events.

## 2. Methods

### 2.1. Waveguide System Test

**Figure 1:**
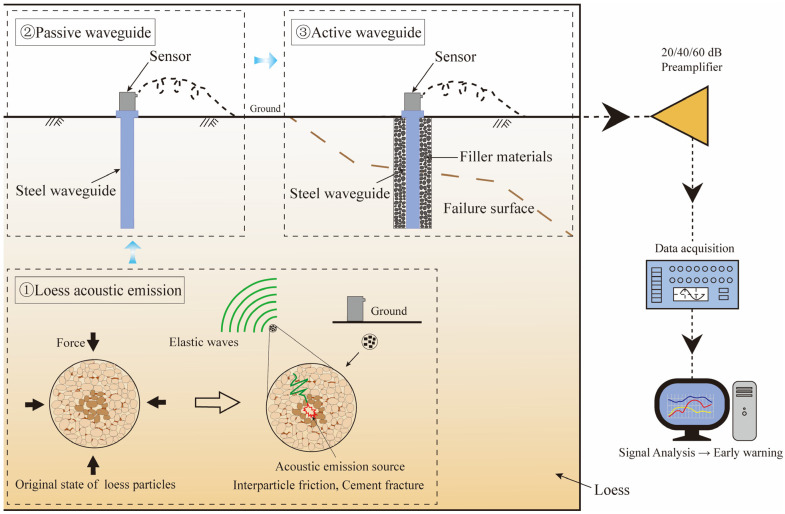
Acoustic emission transmission process.

At present, the two main waveguide modes for AE signals are active waveguide and passive waveguide. Figure 1 shows the transmission process of the AE signal in soil. The passive waveguide system comprises only a waveguide rod and a sensor, where AE signals generated by soil damage propagate directly along the waveguide rod to the sensor. By contrast, the active waveguide system comprises a waveguide rod, a transducer, and a filler material surrounding the waveguide rod. As the soil body deforms, the filler material undergoes compression and shear misalignment, causing collisions with the waveguide rod that generate AE signals. These signals then propagate from the waveguide rod to the sensor.

Active waveguides can effectively monitor soil deformation and failure, addressing the challenges posed by weak AE signal and rapid attenuation. Figure 1 shows that the main factors affecting the effectiveness of the active waveguide system include the sensor installation method, filling materials, waveguide rod wall thickness, wrapping material, and wall thickness. Previous research has not comprehensively analyzed these factors, particularly in relation to the geological hazards associated with loess collapse. This study presents a clear and concise structure with a logical flow of information, focusing specifically on these factors and their impact on filling densities.

#### 2.1.1. Experimental Design

The active waveguide model comprises a waveguide rod, a sensor, and a filler material surrounding the waveguide rod. When soil deformation compresses and causes friction against the filling material, the filling material undergoes compression and shear displacements, colliding with the waveguide rod to generate AE signals that propagate to the sensor through the rod. This model generates AE signals from the filling material. Research indicates that large-particle backfill materials produce the highest response amplitude for active waveguide rods. Additionally, using large particles with high angles and rough surfaces [23] enhances signal generation, while steel wave sensing rods demonstrates strong signal transmission capabilities when compared under the same diameter and length conditions [28]. However, current research on waveguide models does not provide a comprehensive system that determines the optimal selection of materials, specifications, and sensor installation modes. Most existing studies have failed to analyze relevant factors and their correlations adequately. For example, while AE signals can be effectively received with waveguide wall thicknesses of 2, 3, 4, and 5 mm [22,28], different sensor mounting methods, either on the top or on the side of the waveguide, have also been explored [28,29]. However, no consensus on which conditions yield the highest quality AE signals is available. Therefore, developing a reliable AE waveguide system suitable for monitoring loess collapse is urgent.

The experimental tests involved assessing various sensor installation methods, followed by evaluating four types of filling materials: granite, marble, angular gravel, and quartz sand. Table 1 displays the fundamental characteristics of the filler materials used in the tests. Table 2 shows the sequence in which the influences were identified, using a steel waveguide rod with a diameter of 20 mm and wall thicknesses ranging from 2 mm to 5 mm. The outer wrapping materials included high-density polyethylene geomembrane and rubber tubing, with wall thicknesses of 1, 2, and 3 mm. The experiment also tested different filling densities, ranging from 50% to 70%.

#### 2.1.2. Experimental Process

**Figure 2:**
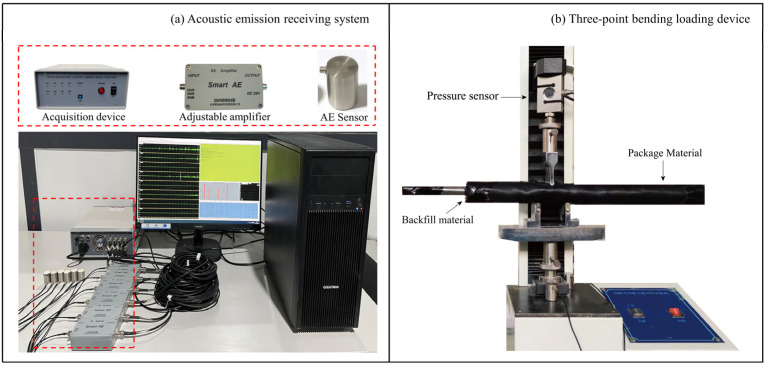
Experimental device.

The servo universal testing machine and DS5-8B AE signal analyzer (Beijing Softland Times Technology Co., Ltd., Beijing, China) were used for testing (Figure 2). The AE waveguide model is placed on the loading equipment. The two ends of the wrapping materials are then closed to prevent the loss of filling materials during the test, and the test displacement is controlled at 1 mm/min.

### 2.2. Collapse Modeling Tests

#### 2.2.1. Test Apparatus

**Figure 3:**
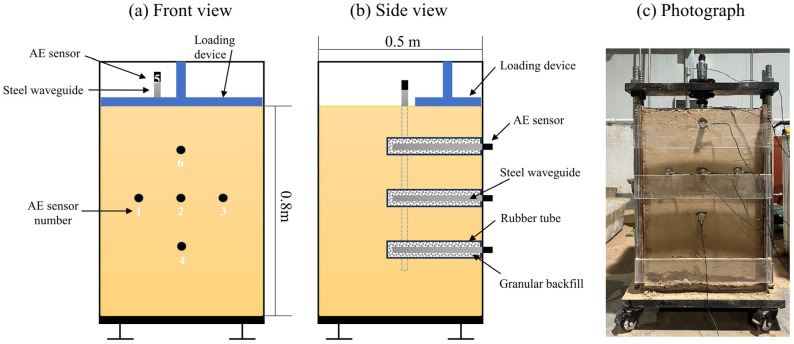
Test systems and devices. In (**a**,**b**), the blue part indicates the pressurized device, and the yellow part indicates the constructed remodeled soil model; (**c**) illustrates the collapse model test. The DS5-8B AE device from Beijing Softland Times Technology Co., Ltd., Beijing, China, was used in this test (Figure 2). This device can acquire and display AE signal waveforms and parameters in real-time. Each channel comprises an AE transducer, a pre-gain adjustable amplifier, an acquisition instrument, and an AE analyzer. Common parameters measured in AE testing include amplitude, ringing count (RDC), and energy. Amplitude represents the maximum height of the AE waveform, while RDC indicates how many times the AE signal exceeds a pre-set voltage threshold within a given time period. Energy corresponds to the area under the signal detection envelope. The AE signals generated by deformation damage in the soil body typically have frequencies greater than 20 kHz. The threshold value of the AE system is set at 45 dB to eliminate the influence of external noise. The AE transducer used is the RS-14A, which has a frequency range of 20–90 kHz, a center frequency of 50 kHz, and a sampling frequency of 2.5 MHz.

A collapse test system was designed using a model box, a loading device, and an AE monitoring device, as shown in Figure 3. The system features a simple structure, high repeatability, and low interference noise during testing. The testing apparatus comprises multiple acrylic panels, with dimensions of 0.5 × 0.6 × 0.8 m, positioned on the base of the loading device. The front acrylic panels of the model apparatus can be opened, allowing for direct observation of the model’s deformation during testing. A hydraulic jack was used to apply thrust, simulating the vertical pressure that could lead to loess collapse. The jack was secured to steel columns on both sides of the model test box, with the thrust directed perpendicular to the bottom of the box. Pressure sensors were also installed on the jacks to measure pressure changes throughout the test, and the data were displayed and recorded in real-time.

#### 2.2.2. Experimental Construction of Loess Collapses Physical Model

(1) Model building

The soil samples were crushed and dried for 12 h. Subsequently, the dried soil was broken down using a crusher and mixed with loess to achieve the desired water content. The amount of water required for the model was calculated using Equation (1) [30], as follows:

(1) $$m_{w}=\frac{m_{0}}{1+0.01\omega_{0}}\times 0.01(\omega_{1}-\omega_{0}),$$

where $m_{w}$(g) indicates the mass of additional water required for the preparation of the model; $m_{0}$(g) represents the mass of the dried soil; $\omega_{0}$(%) refers to the water content of the dried soil; and $\omega_{1}$(%) denotes the water content required for model preparation.

When configuring the water content to prevent loess from caking, water must be added gradually while stirring constantly to ensure uniform distribution without concentration in any area. Once the desired water content is achieved, the mixture is covered with plastic wrap and stored for 12 h. Five random samples are then taken to verify even water distribution.

The model is divided into eight layers, each 10 cm thick after compaction. The mass of loess required for each layer is calculated based on the required dry density for the test. The properly moistened loess is then poured into the model box and compacted using a tamper (25 cm × 25 cm), with each layer compacted three to four times. Any loose material between layers is scraped off prior to tamping to ensure quality. After compaction is completed, ring knife samples are taken to verify that the density meets the required specifications for the test.

(2) Loading method

The AE signal generated during loess damage is weak. Thus, manual pressurization is conducted to exclude the influence of external noise on the test, ensuring a smooth operation of the pressurized oil pump. This approach guarantees that the hydraulic jack gradually applies the force load.

(3) Waveguide system layout

During model construction, the waveguide rods were buried to ensure close contact with the loess, facilitating strong AE signals. Figure 3 shows the six waveguide rods used in the test. All rods were active, except for No. 5, which served as a passive waveguide. The distance between neighboring active waveguide rods was 15 cm. Before conducting the test, the AE system was connected, adjusted, and calibrated. The active waveguide system was constructed based on the test parameters established during the waveguide system tests.

### 2.3. AE Signal Analysis Method

The AE signal parameter analysis method uses multiple simplified waveform characteristic parameters, with time *t* as the horizontal coordinate, to represent AE signal characteristics for further analysis. This approach is straightforward and allows for a comprehensive application of AE parameters, making it reasonable to evaluate the stability of materials or structures and thereby improving the accuracy of AE signal processing. Simultaneously, AE parameter analysis is a relatively mature technology that can process AE signals rapidly, intuitively, and efficiently in real time. Moreover, AE parameter analysis has evolved from the original single-parameter analysis to multiparameter analysis, exhibiting its flexibility. Commonly used AE characterization parameters include the number of AE events, AE ringing count, amplitude, and rise time. The ringing count represents the total amount and frequency of AE activity, making it a useful parameter for evaluating AE activity throughout the monitoring process. Therefore, the AE ringing count is selected for analyzing the AE signals during the tests of the waveguide system and the loess collapse model.

## 3. Results

### 3.1. Waveguide System Test Results

**Figure 4:**
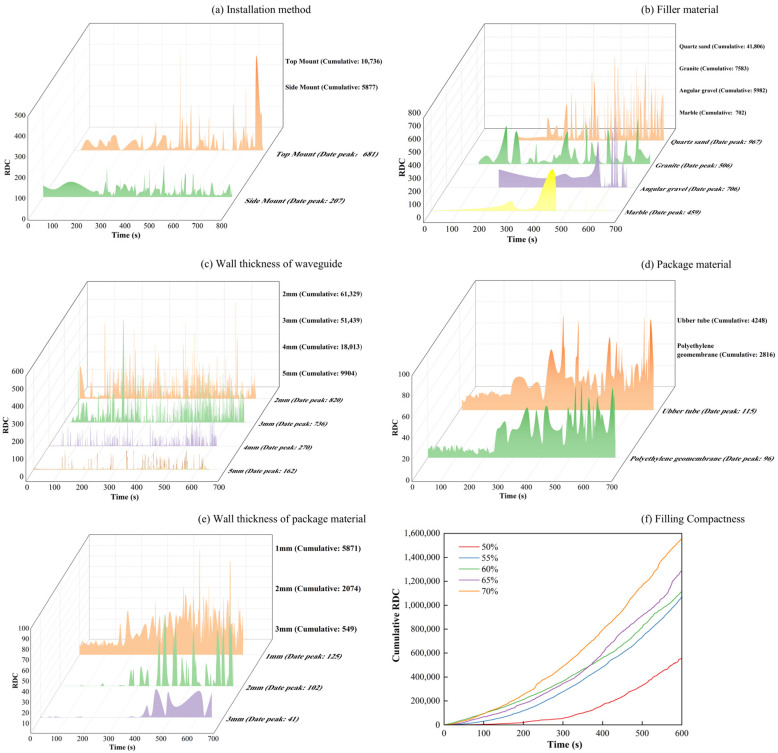
Parameters of active waveguide systems.

According to Figure 4a, the cumulative ringing count of the top-mounted transducer is 10,736 while that for the side-mounted transducer is 5877. At the start of the loading test, AE signals were detectable by either the top-mounted or side-mounted transducer, with similar trends observed for each mounting method. However, the response trend from the top-mounted transducer became more pronounced after a certain period of loading. Regarding the filling materials (Figure 4b), AE signals from quartz sand and granite were more pronounced and detected earlier. The cumulative ring counts for quartz sand, granite, angular gravel, and marble were 41,806, 7583, 5982, and 702, respectively. The signals generated by quartz sand exhibit a more pronounced response during loading, particularly due to AE signals arising from extrusion, friction of the filler material, and shear misalignment. The waveform parameters of these signals are notably larger. Regarding the steel waveguide wall thickness (Figure 4c), the AE signal is most evident with a wall thickness of 2 mm, where the cumulative ring counts reach 61,329, 51,439, and 18,013 for 2, 3, and 4 mm wall thicknesses, respectively. The cumulative AE ring count is 9904 for a wall thickness of 5 mm. The AE signals obtained from the four different wall thicknesses exhibit similar trends throughout the loading process. In terms of wrapping materials (Figure 4d), the rubber tube and the polyethylene geomembrane produce distinct AE signals. The rubber tube has a cumulative ring count of 4248, while the polyethylene geomembrane has 2816. The response trend of the AE signals obtained from both types of wrapping materials remains consistent during the loading process. For the wall thickness of wrapping materials (Figure 4e), the AE signals are most pronounced when the rubber tube has a wall thickness of 1 mm. The cumulative AE ring counts are 5871, 2074, and 549 for wall thicknesses of 1, 2, and 3 mm, respectively. Figure 4e shows the response trend of the AE signal parameter for the rubber tube with a 1 mm wall thickness throughout the loading process, showing consistently high magnitude values. The AE signal parameter for the 1 mm thick rubber tube exhibits the most notable response trend during loading, with a markedly larger magnitude compared to the other wall thicknesses.

An active waveguide system has been developed for monitoring AEs in loess collapse geological disasters. This system comprises a stainless-steel waveguide rod, an AE sensor, a rubber tube sleeve, and a backfill material. The AE sensor is mounted on top of the waveguide rod, which has a diameter of 20 mm and a wall thickness of 2 mm. The rubber tube sleeve has a diameter of 52 mm and a wall thickness of 1 mm. The filler material between the waveguide rod and the rubber tube sleeve comprises well-graded prismatic–subprismatic quartz sand particles, with particle sizes ranging from 3 mm to 20 mm. Subsequently, three-point bending tests were conducted at different filling densities: 50%, 55%, 60%, 65%, and 70%. Figure 4f shows that the cumulative RDC values for densities of 60%, 65%, and 70% are similar in the first 200 s. However, the AE data for 50% and 55% densities are drastically lower than those for the other densities. As the test force increases, the amount of AE signals received for higher densities also rises. At 600 s, the amount of AE signals received for 50% densities is only 35.7% of that received for 70% densities.

### 3.2. Collapse Modeling Tests Results

#### 3.2.1. Forms of Collapse Damage

**Figure 5:**
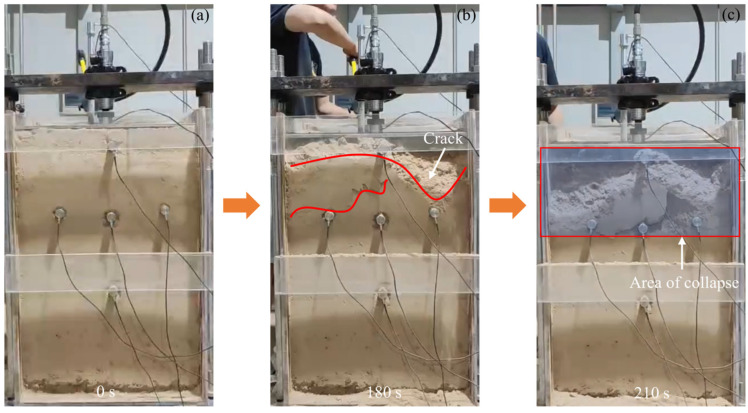
Collapse model test procedure. (**a**–**c**) represent the process by which the model was destroyed during the experiment.

Figure 5a illustrates the initiation status of the model test. Figure 5b shows that the upper part of the model began to gradually collapse at 180 s, with a notable loess collapse occurring above sensor #3 and a crack forming near sensor #1. At 210 s, the test force peaked at 2480 kPa, resulting in extensive collapse of the upper part of the model, as shown in the rectangular range in Figure 5c. The main collapse areas were located near sensors #1, #2, and #3.

#### 3.2.2. AE Signal Characterization

**Figure 6:**
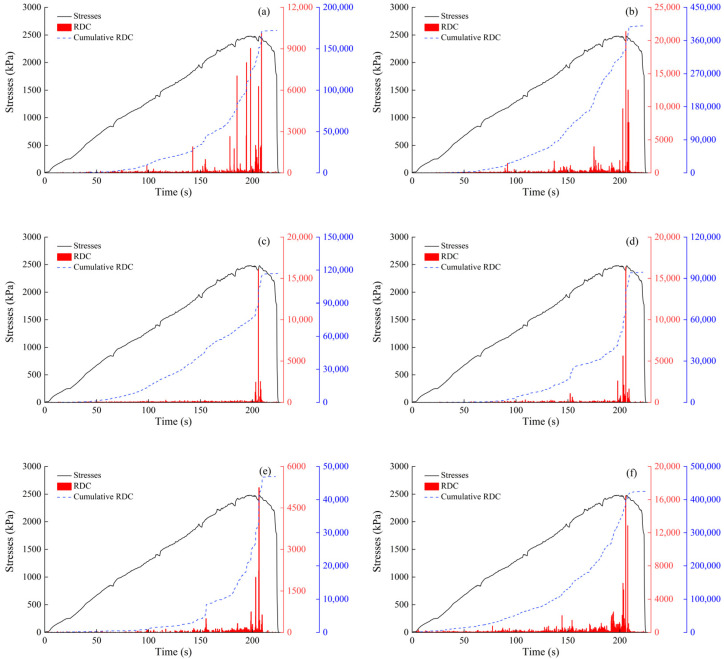
Test stresses–RDC, cumulative RDC relationship. (**a**) Sensor #1; (**b**) sensor #2; (**c**) sensor #3; (**d**) sensor #4; (**e**) sensor #5; and (**f**) sensor #6.

**Figure 7:**
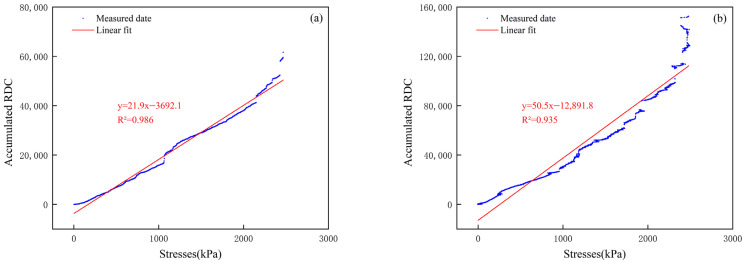
Linear relationship between cumulative AE and stress. (**a**) Sensor #4; and (**b**) sensor #1.

Figure 6 shows a large AE ring count recorded by transducer No. 1 during the 180 s test, indicating the possible formation of a large fissure in the soil above the transducer. The AE signal intensity received by transducer No. 5 was lower than that of the other transducers, with peak and cumulative AE ring counts of 5237 and 46,951, representing only 24.49% and 11.74%, respectively. The passive waveguide has a lower capacity to receive AE signals compared to the active waveguide. Figure 7 shows that the fitted curve of the cumulative AE ring counts collected by sensor #1 is expressed as y = 50.5x − 12,891.8, with an R^2^ value of 0.935, indicating excellent positive correlation. The fitted curve of the cumulative AE collected by sensor #4 also shows excellent positive correlation (R^2^ = 0.986). The degree of extrusion and friction between the filler particles increases with pressure throughout the collapse process. Intense friction and extrusion result in louder emission signals.

## 4. Discussion

As demonstrated in Figure 4, the intensity of the AE signal increases with the compactness of the filler material. However, an upper limit to the compactness of the filling material is observed during actual testing. Liu et al. (2020) [2] found that the range of quartz sand porosity ratio is approximately 0.3 to 0.8, corresponding to a compactness range of 56% to 77%. When the diameter ratio is 0.25 and the volume fraction of fine particles is 0.19, the minimum porosity ratio of the quartz sand is approximately 0.54, corresponding to a maximum compactness of approximately 65%. The pore ratio of the filler material decreases with the diameter ratio, resulting in greater filler compactness. The quartz sand filler material used in this study has a diameter ratio of approximately 0.19 and a fine particle volume fraction of approximately 0.19, achieving a maximum compactness of 70%.

Variations in acoustic signal strength produced by the transducer mountings are attributed to coupling attenuation. The waveguide rod has a slot at the top, resulting in a larger contact area between the top-mounted transducer and the waveguide rod than the side-mounted one. Therefore, the top-mounted transducer can acquire strong AE signals when mounted at the top due to a tight connection.

The strength of the AE signal produced by different wrapping materials differs due to geometric diffusion and coupling attenuation. The properties of the rubber tube must be considered during the selection of the outer wrapping material. While this tube is softer and more sensitive to external changes, it can be wrapped more tightly with the filler material. In addition, the rubber tube limits geometric expansion of the acoustic wave along the direction of attenuation, resulting in a strong AE signal. The language used in this text is clear, objective, and value neutral, adhering to the desired characteristics.

The strength of the AE signal produced by waveguide rods and rubber tubes varies with their wall thickness due to material absorption attenuation. Thick materials absorb additional energy as the acoustic wave source expands in all directions, resulting in acoustic signal attenuation. Therefore, thin waveguide rods and rubber tubes produce strong acoustic signals.

**Figure 8:**
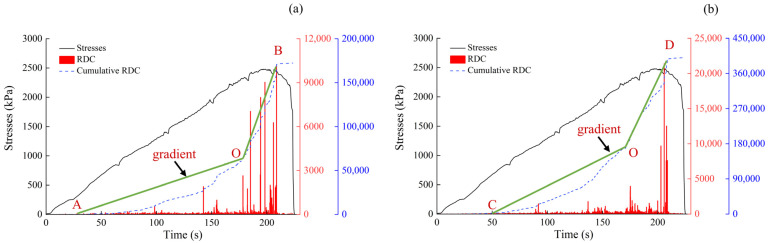
Cumulative RDC slope change. (**a**) Sensor #1; and (**b**) sensor #2.

Figure 8a shows almost no AE ring counts in the early stage of the test, but they gradually appeared at 50 s. At 178 s, a sudden increase in ringing counts was observed, which also gradually increased until reaching its peak. The green line segment in the figure indicates the change in the slopes of the cumulative ring counts before and after the sudden change. Figure 8a shows that the slope of segment OA was 504, whereas that of segment OB was 2387, representing an increase of 474%. Moreover, the time point of the sudden change in slope occurred 32 s before the collapse. As shown in Figure 8b, the ringing counts suddenly increased at 175 s. The slope of the OC segment before the sudden change was 1595, and that of the OD segment after the sudden change was 4081, representing an increase of 256%. The time point of the sudden change in the slope was 35 s earlier than the collapse. The AE ringing counts exhibit a strong correlation with the loess collapse process, effectively reflecting this procedure.

As an important parameter for investigating the failure mechanical mechanism of the loess collapse, b-values can be used to reflect changes in microfracture scale inside the loess collapse. A decrease in b-value indicates an increase in the proportion of large events inside the loess collapse, that is, the number of large-scale microfractures notably increases [31]. The results indicate that when the b-value is large, the AE signal amplitude collected in the corresponding sampling window is low, suggesting that the vibrations generated by AE events during the loess collapse are weak, primarily resulting in small cracks. Conversely, when the b-value is small, the AE signal amplitude is high, indicating stronger vibrations from AE events in the loess collapse, which are generally associated with larger cracks. If the b-value remains unchanged over a period, then the size of AE events also remains constant, indicating that the fracture scale within the sample is relatively stable. Conversely, when the b-value exhibits small amplitude changes, the fracture scale within the sample undergoes abrupt changes. Substantial changes in b-values indicate a transient mutation state of loess collapse [32,33]. Therefore, changes in the b-value can serve as an important precursor for monitoring damage associated with loess collapse. The concept of the b-value was first introduced in seismology, where researchers described the relationship between decreasing earthquake frequency and magnitude (Gutenberg et al., 1994) [19] and proposed the following empirical Formula (2):

(2) log⁡N=a−bM.

When calculating the b-value for the AE of rock damage, the amplitude of the AE is generally used to indicate the size of events. The b-value size denotes the proportion of low-amplitude events to high-amplitude events. The maximum likelihood method is employed to calculate the b-value of the AE associated with rock damage, using Formula (3), as follows:

(3) $$b=\frac{20\times \log e}{\left(\bar{A}-A_{min}\right)}$$

**Figure 9:**
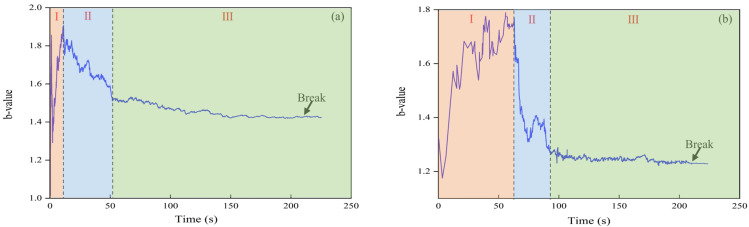
Variation of b-values for the active waveguide system. (**a**) Sensor #1; and (**b**) sensor #2.

Figure 9 illustrates the three stages of the b-value: rising, falling, and steady periods. Stage I presents a rising trend in the b-value. Stage II exhibits a gradual appearance of high-amplitude signals in the AE, and the b-value experiences a rapid decline. In stage III, a substantial number of high-amplitude AE signals are detected, and the b-value exhibits a steady decline. The b-values of sensors #1 and #2 enter the steady period 117 and 143 s earlier than the collapse of the soil body, respectively. However, while the b-value can provide an early warning for loess collapse, it should be verified through further field experiments, considering the complex working conditions of actual loess slopes.

Overall, the study found that AE signal strength is related to the density of the filling material; higher density corresponds to greater signal strength. However, this density has an upper limit, influenced by the pore and diameter ratios of the quartz sand. When the diameter ratio and fine particle volume fraction remain constant, the pore ratio of quartz sand decreases as density increases, but this relationship plateaus after reaching a certain degree. In this study, the maximum density of quartz sand filling material was determined to be 70%. Additionally, the sensor’s installation affects signal strength due to coupling attenuation. A top-mounted sensor, having a larger contact area with the waveguide rod, achieves a stronger AE signal. The intensity of AE signals produced by different wrapping materials is different. The rubber tube can be more tightly packed with the filling material due to its softness and sensitivity to external changes, and the geometric diffusion of the sound wave along the attenuation direction is smaller, resulting in stronger AE signals. The wall thickness of the waveguide rod and rubber tube will affect the AE signal intensity through material absorption attenuation. Thick wall materials absorb additional energy, resulting in acoustic signal attenuation. Therefore, thin waveguide rods and rubber tubes can produce strong acoustic signals. The AE ringing count has a strong correlation with the collapse process of loess, effectively reflecting the process. In the test, the ringing count suddenly increases before the collapse, and the change in slope and the collapse time demonstrate certain advancements, indicating that the ringing count can be used as the precursor information of loess collapse. The b-value of sensors #1 and #2 entered the stable period 117 and 143 s before the soil collapse, respectively, indicating the crucial role of the b-value in loess collapse.

## 5. Conclusions

This study investigates and designs an AE waveguide system for loess. The system encompasses the installation method of the sensor, the selection of the filler particle material, the wall thickness dimension of the steel waveguide rod, the selection of the wrapping materials and the wall thickness dimension, as well as different filler densities. Tests were conducted on an indoor loess collapse model, and the AE waveguide system was preliminarily verified for its application in loess collapse. The main conclusions are presented as follows:

(1) The system comprises an active waveguide made up of waveguide rods, thin-walled wrapping materials, and high-density filler materials. This system can produce a substantial amount of AE signals, which can improve the quality of AE data acquisition in monitoring loess collapses.
(2) Strong correlation was found between cumulative AE ring counts and stress during indoor modeling tests. Moreover, a strong synchronization was observed between RDC and the loess damage process. These findings indicate that AE ring counts can accurately quantify the damage process of loess collapse.
(3) Analysis of the AE parameters reveal a sudden change in the slope of the cumulative AE ring counts before the imminent collapse of the loess. This phenomenon occurs during the smooth period of the b-value. This finding indicates that the slope of the cumulative AE ring counts and the trend of the b-value may serve as precursor information for early warning of loess collapse.

Some problems identified in the research process must also be addressed. First, while the factors affecting the AE signal strength of the active waveguide system are investigated, other factors may have been overlooked, which may also affect the monitoring and early warning of loess collapse. Second, although the study proposes that the b-value can provide early warning for loess collapse, this conclusion must be verified by further field experiments due to the complex working conditions of the actual loess slope. Relevant field experimental data support is also lacking. Furthermore, the results of this paper are mainly based on laboratory model tests due to the scale effect, which may require further validation and adjustment, considering complicated situations in real engineering.

## Figures and Tables

**Table 1:** Basic parameters of filling materials.

Name	Sizes (mm)	Hardness	Aspect Ratio	Roundness	Convexity	Densities
Quartz sand	3–20	7	1.62	1.95	0.96	2.52
Granite	3–20	6–7	1.72	1.80	0.89	2.78
Angular gravel	3–20	4–5	1.75	1.52	0.97	2.57
Marble	3–20	3–4	1.72	2.27	0.95	2.62

**Table 2:** Experimental design.

Step 1. Determine the installation method	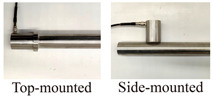
Step 2. Determine the filler material	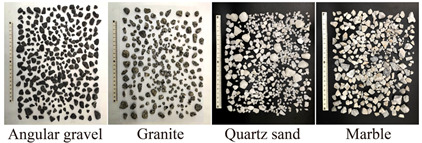
Step 3. Determine the steel waveguide wall thickness	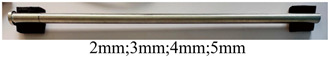
Step 4. Determine the package material	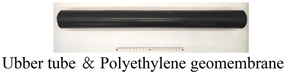
Step 5. Determine the package material wall thickness	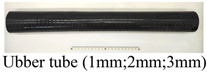
Step 5. Determine the filling compactness	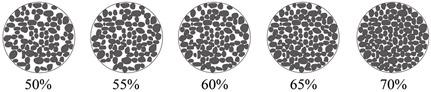

## Data Availability

Data are contained within the article.
